# A Mesoscopic Traffic Data Assimilation Framework for Vehicle Density Estimation on Urban Traffic Networks Based on Particle Filters

**DOI:** 10.3390/e21040358

**Published:** 2019-04-03

**Authors:** Song Wang, Xu Xie, Rusheng Ju

**Affiliations:** College of Systems Engineering, National University of Defense Technology, Changsha 410073, China

**Keywords:** data assimilation, vehicle density estimation, platoon based model, event-based data, particle filters

## Abstract

Traffic conditions can be more accurately estimated using data assimilation techniques since these methods incorporate an imperfect traffic simulation model with the (partial) noisy measurement data. In this paper, we propose a data assimilation framework for vehicle density estimation on urban traffic networks. To compromise between computational efficiency and estimation accuracy, a mesoscopic traffic simulation model (we choose the platoon based model) is employed in this framework. Vehicle passages from loop detectors are considered as the measurement data which contain errors, such as missed and false detections. Due to the nonlinear and non-Gaussian nature of the problem, particle filters are adopted to carry out the state estimation, since this method does not have any restrictions on the model dynamics and error assumptions. Simulation experiments are carried out to test the proposed data assimilation framework, and the results show that the proposed framework can provide good vehicle density estimation on relatively large urban traffic networks under moderate sensor quality. The sensitivity analysis proves that the proposed framework is robust to errors both in the model and in the measurements.

## 1. Introduction

Traffic state information, such as the density, speed on road segments and the queue size in front of an intersection, is the basis of various road traffic management and control strategies. They range from traffic light control [1], ramp metering [2] to link control [3], and route guidance [4]. Estimation of the traffic state is necessary due to the limited coverage of sensors and to the noisy measurements that the sensors produce [5]. Traffic models and traffic simulations play an important role in traffic engineering and traffic control and are widely used in traffic state estimation [5,6].

However, many factors influence the accuracy of traffic simulation results. Firstly, since every traffic flow model is a simplification of a real traffic system which is complex and uncertain in nature, errors from the process of modeling are inevitable. They include both the inaccurate modeling, the errors in parametric data as well as the uncertainty in traffic systems [7,8,9,10]. Moreover, unpredictable traffic events, such as automobile accidents, make the estimate of traffic simulations far from the real traffic condition. In order to reduce these errors and improve the accuracy of traffic simulation results, data assimilation techniques are employed.

Data assimilation aims to incorporate the observed information into the dynamic system model to produce improved state estimates [11,12] where the three elements of system model, measurement model and data assimilation algorithm are involved. It has been widely applied in areas such as atmosphere, ocean climatology and hydrology [13,14,15]. In attempts to estimate the traffic state, efforts have been made to assimilate traffic data into traffic flow models. For example, Yuan et al. [16,17] employ the extended Kalman filter (EKF) to assimilate flow, speed data and floating car data into a Lagrangian macroscopic traffic flow model in order to estimate the traffic density and speed on freeways. However, EKF can be applied only when the traffic flow model is differentiable with respective to its state, therefore it excludes many traffic models including the Cell Transmission Model (CTM) [18] which is widely used in macro-level traffic simulation [19]. In order to avoid this problem, Thai and Bayen [20] transform the CTM to a switching mode model (SMM) in which the system switches between some linear models and incorporates sparse density observations from loop detectors into it using Kalman filter (KF) techniques to estimate densities on a highway. The ensemble Kalman filter (EnKF), which can address non-differentiable system models but is restricted to Gaussian model errors, is applied to assimilate the GPS speed and position data into a velocity based macroscopic traffic model to estimate the mean speed on a highway in [21]. While most data assimilation algorithms are limited by the assumptions of either linear/continuously differentiable models (e.g., KF, EKF) or Gaussian errors (e.g., EnKF), the particle filter (PF) imposes no restriction on the dynamics and the errors of the models and can converge to the true state distribution. It is superior for the estimation of nonlinear and non-Gaussian system theoretically [22] and has been employed in many related research works [23,24]. Xie et al. [24] develop a generic particle filter-based data assimilation framework for reconstructing vehicle trajectories on signalized urban arterials, in which noisy vehicle passages and sparse travel time observations are assimilated into a microscopic traffic simulation model.

The objective of our study is to estimate vehicle densities on the roads in an urban traffic network. Vehicle density is one of the main variables describing the urban traffic state and provides important information for urban traffic control, such as the control of traffic lights [1], and vehicular traffic guidance [25]. Three classes of traffic simulation models can be used to perform the estimation of vehicular traffic density. Microscopic traffic models describe the movement of each individual vehicle in detail. Macroscopic traffic models describe the spatial-temporal evolution of aggregated traffic variables. Mesoscopic traffic models combine microscopic and macroscopic aspects of traffic flow dynamics in some forms. On the one hand, mesoscopic traffic models are computationally faster and more appropriate in large urban traffic networks than microscopic traffic models. On the other hand, mesoscopic traffic models keep track of more details compared with macroscopic traffic models. Therefore, we propose a novel data assimilation framework to estimate vehicle densities on relatively large urban networks. The novelty is twofold. Firstly, it makes a good compromise between model details and computational cost by using a mesoscopic traffic model. Secondly, this framework integrates informative event-based data which is rarely used in related research. In this mesoscopic traffic data assimilation framework, the platoon based model [26] is employed since it not only explicitly captures the heterogeneity (the gap between successive platoons) characterizing urban traffic but also is computationally efficient. Vehicle passage times from sensors (e.g., loop detectors) are considered as the measurement data, which contains errors such as missed and false detections. Since the mesoscopic traffic model is nonlinear and the measurements of vehicle passage times contain strongly non-Gaussian noises, we employ particle filters to conduct the data assimilation due to its advantages mentioned above. We conduct simulation experiments to test the data assimilation framework, and the results show that this method can provide reasonable estimates of vehicle density under moderate sensor quality. Further sensitivity analysis indicates its robustness to errors both in the model and in the data.

The rest of this paper is structured as follows. In Section 2, we formally present the mesoscopic traffic model with the Discrete Event System Specification (DEVS) formalism due to its discrete event nature. Then, Section 3 presents the particle filter based mesoscopic traffic data assimilation framework for vehicle density estimation on urban traffic networks. The results of experiments and sensitivity analysis are presented in Section 4. Finally, conclusions are drawn in Section 5.

## 2. Mesoscopic Urban Traffic Model in the DEVS Formalism

Previous research has defined and validated the approach of aggregating vehicles into platoons in the urban traffic through analyzing real measurements [26]. Since platoon based model (PBM) makes a good compromise between computational efficiency and simulation accuracy, we employ it as our traffic flow model with the expectation that our proposed data assimilation framework can be applied in relatively large urban traffic networks.

The PBM is a typical discrete event system model, so we formally describe it using the DEVS formalism [27] which is widely adopted in discrete event modeling and simulation. Firstly, we identify the atomic components of an urban traffic system and present their coupling relations to construct a network. Then, we depict the dynamic behaviors of some key atomic models with the DEVS formalism.

### 2.1. The Coupled DEVS Model of the Urban Traffic System

Conceptually, an urban traffic network is composed of links and intersections with specific origins and destinations of traffic demands. Following the DEVS framework, an urban traffic network is represented as a coupled model which consists of atomic components. We identify six types of atomic components in an urban traffic system:

- source model *A*, which randomly generates platoons of vehicles according to the traffic arrival flow and sends them into the urban traffic network;
- segment model *M*, which represents either a section of road links *S* or a preselection lane *P* at the entrance of a intersection and describes the movement of vehicle platoons on it;
- assignment model *D*, which randomly assigns platoons that will enter an intersection to the preselection lanes according to the given turning probabilities;
- intersection model *I*, which imitates the behavior of a physical intersection in urban traffic networks and transfers platoons from the preselection lanes at entrance points to the exit links;
- traffic light model *L*, which sends index signals to an intersection model to switch the phase of traffic light periodically. In our study, the fixed-time traffic light control is employed;
- sink model *B*, which serves as the destination of vehicles and records information of platoons leaving the network under study.

For an urban traffic network under consideration, we define a set {So,Sg,Ag,Int,Tl,Sk} to categorize all related atomic components, where So is the set of all related source models (i.e., $So=\{A_{i},i=1,\ldots ,N_{A}\}$), Sg is the set of all related segment models (i.e., $Sg=\{M_{i},i=1,\ldots ,N_{M}\}$), Ag is the set of all related assignment models (i.e., $Ag=\{D_{i},i=1,\ldots ,N_{D}\}$), Int is the set of all related intersection models (i.e., $Int=\{I_{i},i=1,\ldots ,N_{I}\}$), Tl is the set of all related traffic light models (i.e., $Tl=\{L_{i},i=1,\ldots ,N_{L}\}$), and Sk is the set of all related sink models (i.e., $Sk=\{B_{i},i=1,\ldots ,N_{B}\}$).

**Figure 1:**
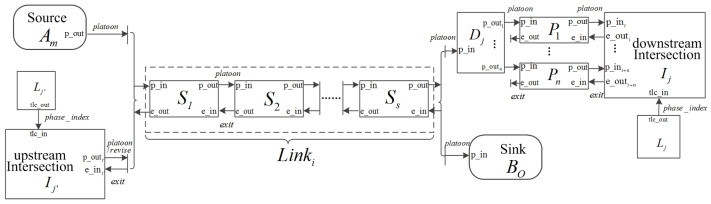
The coupled DEVS model of an urban traffic network.

In addition, four types of messages which are transmitted between atomic models are defined:

- platoon message, representing a group of vehicles traveling together with the same speed (i.e., the platoon of vehicles). The platoon message is characterized by variables ($T_{head}$, $P_{size}$), indicating the time instant when the head of the platoon arrives at the entrance boundary of the current segment/intersection and the number of vehicles within the platoon respectively;
- exit message, used to block (exit=0) or free (exit=1) the exit boundaries of segment models (maybe via an intersection model);
- revise message, used to revise the number of vehicles on the downstream segment when a platoon is split by the red traffic light. The platoon messages and revise messages are transmitted to a segment model via the same port. A revise message consists of variables ($flag_{r}$, $N_{r}$), where $flag_{r}$ is used to distinguish the revise message from the platoon message (for example, $flag_{r}=-1$ when $T_{head}\geq 0$ in platoon messages are assured in a simulation) and $N_{r}$ indicates the number of vehicles failing to cross the stop line.
- phase_index message, which indexes the phase of the traffic light and is sent to an intersection model by a traffic light model;

Figure 1 illustrates how the atomic models form a coupled urban traffic network model using ports. In Figure 1, the rectangles represent atomic models with input and output ports and the arrows show the connections where messages are sent from an output port to an input port of models. A road link $Link_{i}$ can be represented by a sequence of segment models (donated as $S_{1},\ldots ,S_{s}$) where platoon messages are transmitted from the upstream to the downstream segment and exit messages are transmitted from the downstream to the upstream segment. The first segment $S_{1}$ receives platoon messages from an upstream source $A_{m}$ or Intersection $I_{j}^{\prime }$. The last segment $S_{s}$ sends platoon messages to the downstream component. If the downstream component is a sink model, platoons can enter it directly. Otherwise, the downstream of this link is connected to an intersection. In this case, $S_{s}$ first sends platoon messages to an assignment model $D_{j}$ in order to assign the vehicles within a platoon to different preselection lanes. Then, the platoons are sent to an intersection model $Int_{j}$ by the preselection lanes. The exit messages are transmitted from the intersection model $Int_{j}$ to $S_{s}$ via their preselection lanes. Intersection models transmit platoon and revise messages to the downstream links and receive exit messages from them. For each intersection, there is a corresponding traffic light model which sends phase_index messages to it. Notice that the coupled urban traffic network model has no external input and output.

### 2.2. Key Atomic Components of the Urban Traffic System

In this subsection, we will describe the atomic models of source, segment, and intersection in detail. Each atomic component is modeled into different phases. The phase variable qualitatively partitions the infinite state space into finite mutually exclusive and collectively exhaustive subsets (i.e., phases) where the dynamics of atomic models are recognizable. Thus, we can specify the behavior of atomic models (e.g., the time advance, transition, and output function) in each phase. Phases make models more understandable, validatable, and communicable [28]. The phases and state variables of these atomic components are listed in Table 1. Since the other models (i.e., sink model, assignment model and fixed-time traffic light model) are quite simple, we omit them in this paper due to the limited space.

#### 2.2.1. Source Model

As a DEVS model, the source model remains active all the time and creates platoons of vehicles by sending platoon messages to the connected link continuously based on a vehicle arrival rate.

Let the n-th platoon message be sent out at time $p\_time_{n}$ in which the number of vehicles is $p\_size_{n}$, then the time when sending the (*n* + 1)-th platoon $p\_time_{n+1}$ is determined by

(1) $$p\_time_{n+1}=p\_time_{n}+p\_size_{n}\cdot hw+\Delta +r_{gap},$$

where hw is the average time interval between two successive vehicles within a platoon entering the network, Δ is a pre-determined value which represents the minimum time gap between successive platoons, and $r_{gap}$ is an exponentially distributed random variable.

As a result, the vehicle arrival rate *q* is determined by

(2) $$q=\frac{E(p\_size)}{\Delta +E\left(r_{gap}\right)+E\left(p\_size\right)\cdot hw},$$

where $E(r_{gap})$ is the mean value of $r_{gap}$, E(p_size) is the mean value of the size of the platoon generated which is drawn from a binomial distribution with size limit of $p\_size_{max}$. According to Equation (2), given the vehicle arrival rate, E(p_size) is calculated by

(3) $$E\left(p\_size\right)=\frac{q\cdot (\Delta +E\left(r_{gap}\right))}{1-q\cdot hw}.$$

#### 2.2.2. Segment Model

The segment model has two pairs of input and output port: InPorts={“p_in”,“e_in”}, OutPorts={“p_out”,“e_out”}, where “p_in” is used to receive platoon/revise messages, “p_out” is used to send platoon messages, “e_in” and “e_out” are used to get and send exit messages. Three attributes are defined for the segment model: $V_{max}$ is the speed limit of the segment, segLength is the length of the segment, and *C* represents the maximum number of vehicles on the segment. There are three state variables in the segment model: platoonList records the information of all platoons on the segment including the platoon which is entering or leaving the segment; vn is the number of all vehicles in platoonList; out indicates whether the platoons can leave the segment when arriving the boundary.

When a platoon characterized by ($T_{head}$, $P_{size}$) enters a segment, it travels on the segment with an independently random speed $P_{v}=p\cdot V_{max}$, where *p* is a random variable indicating the speed profiles of platoons on urban roads. The same as in [26], we assume *p* = 1.0, 0.9, 0.8 with probabilities of 0.8, 0.15 and 0.05, respectively. Then, the element of ($T_{head}$, $P_{size}$, $P_{v}$) is added to platoonList. Notice that, unlike [26], the queue size is not represented separately in our study, since we focus on the vehicle density on the segment. However, if we need the queue size (e.g., when the vehicles in the queue exit the segment as a single platoon), it can be calculated as in [29]. In the platoon based model, the movements of platoons on the segment are not traced, only the entries and exits of platoons are dealt with, and overtaking of platoons within a segment is not considered currently. If a faster platoon catches up with a slower platoon, they merge as a single platoon.

As is shown in Table 1, eight phases are defined to model the dynamical evolution of an urban road in the segment model:

- empty, which indicates there is no vehicle on the segment (i.e., vn=0);
- approach, which indicates the first platoon in platoonList is approaching the exit boundary of the segment;
- cross, which indicates the first platoon in platoonList is crossing the exit boundary of the segment;
- blocked, which indicates the head of the first platoon in platoonList has arrived at the blocked exit boundary and the segment can contain all the vehicles in platoonList;
- blocked_in, which indicates the head of the first platoon in platoonList has arrived at the blocked exit boundary and the last platoon in platoonList is entering and will totally occupy the segment;
- blocked_full, which indicates the exit boundary of the segment is blocked and the segment is totally occupied by vehicles;
- transient_p, which is a transient phase with 0 time duration. The segment model moves to transient_p in order to output a platoon message;
- transient_e, which is also a transient phase. The segment model moves to transient_e in order to output an exit message.

**Figure 2:**
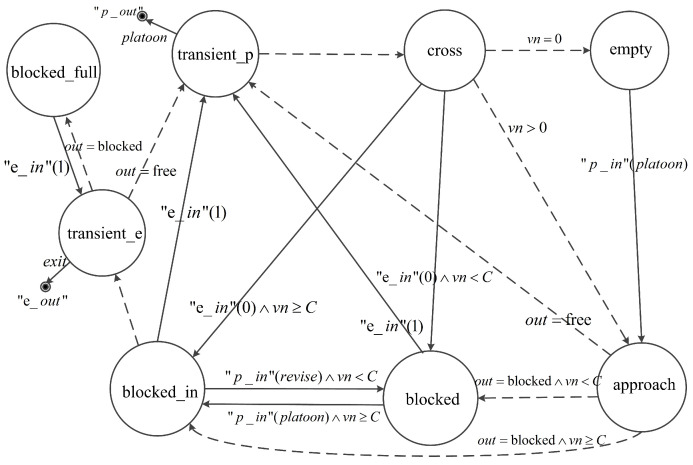
Phase transition graph of the segment model.

Figure 2 shows the phase transitions of the segment model. In the diagram, external transitions and message outputs are represented by solid arrow lines, while internal transitions are represented by the dashed arrow lines. Conditions of transitions are indicated together with the arrow lines representing the internal/external transitions. When a segment is in empty, a phase transition to approach takes place immediately if receiving a platoon message through “p_in”. The phase stays approach until the time when the first platoon reaches the exit boundary. If the boundary is free (i.e., out = free), the phase moves to transient_p to send the platoon message to the downstream model through “p_out” and instantaneously a phase transition to cross occurs. As soon as the platoon leaves the segment completely, the segment removes it from platoonList. In this case, if there still are platoons on the segment (i.e., vn>0), the phase moves back to approach. Otherwise, the phase moves to empty.

The exit boundary becomes blocked if a segment receives a blocked exit message (i.e., exit=0) from “e_in”. If the phase of a segment is approach, a queue forms when the first platoon arrives at the blocked boundary. In this case, the phase transition depends on the number of vehicles in platoonList. If vn≥C, the phase jumps to blocked_in. Otherwise, the phase moves to blocked. If the phase is cross when a segment receives a blocked exit message, the crossing platoon is split, and the phase transition also depends on vn like in the approach case.

In phase blocked, if receiving a platoon message results in excessive vehicles (i.e., vn≥C), the segment also transits to blocked_in. If the segment is full, the phase enters blocked_full via transient phase transient_e for sending a blocked exit message to the upstream model through “e_out”. In phase blocked_full, as soon as a free exit message (i.e., exit=1) is received from “e_in”, the segment jumps to transient_e and transient_p successively in order to send a free exit message to the upstream model and send a platoon message to the downstream model, then the phase enters cross. In the case that the phase is blocked or blocked_in when a free exit message is received, a phase transition to cross via transient_p occurs.

In addition, the segment which is connected to the exit point of an intersection can receive revise messages from “p_in”. In this case, if the segment is in blocked_in and the revised platoon can no longer totally occupy the segment (i.e., vn<C), the phase moves to blocked.

#### 2.2.3. Intersection Model

An intersection connects the upstream preselection lanes and the downstream exit segments. Three types of input ports and two types of output ports are defined in the intersection model. Inports ={${\left\{“p\_in_{m}”\right\}}_{m=1}^{msize}$, ${\left\{“e\_in_{n}”\right\}}_{n=1}^{nsize}$, “tlc_in”}, Outports = {${\left\{“p\_out_{n}”\right\}}_{n=1}^{nsize}$, ${\left\{“e\_out_{m}”\right\}}_{m=1}^{msize}$}, where $“p\_in_{m}”$ is used to receive platoon messages from an upstream preselection lane, $“e\_in_{n}”$ is used to receive exit messages from a downstream exit segment, “tlc_in” is used to receive phase_index messages from a traffic light model, $“p\_out_{n}”$ is used to send platoon/revise messages to a downstream segment, $“e\_out_{m}”$ is used to send exit messages to an upstream preselection lane, and msize, nsize are the number of the upstream lanes and downstream segments, respectively. In an intersection model, each upstream preselection lane $i_{m}$ corresponds to a pair of ($“p\_in_{m}”$, $“e\_out_{m}”$) and Ent represents the set of all preselection lanes (i.e., $i_{m}\in Ent$), while each downstream segment $o_{n}$ corresponds to a pair of ($“p\_out_{n}”$, $“e\_in_{n}”$) and Ext represents the set of all downstream segments (i.e., $o_{n}\in Ext$).

In order to associate the preselection lanes with the exit segments and enumerate phases of the traffic light in an intersection, the following variables are defined:

- ODMap, which maps a preselection lane in Ent to an exit segment in Ext.
- DOMap, which maps an exit segment in Ext to several preselection lanes in Ent.
- TLPhases, which contains all phases of the traffic light in an intersection. The phase of the traffic light is represented by a subset of Ent (i.e., TLPhases(i)⊂Ent, where *i* is the index of the phase), which lists the preselection lanes for which the traffic light is green.

**Figure 3:**
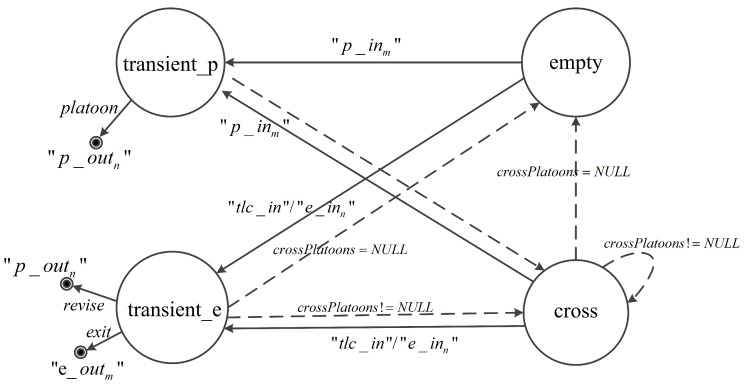
Phase flow graph of the intersection model.

As is shown in Table 1, there are two state variables in the intersection model: crossPlatoons contains the related information of platoons which are crossing the entrance boundary of the intersection; currentPhase records the current phase of the traffic light in the intersection. Four phases evolve in the intersection model: $Phase_{I}=\left\{empty,cross,transient\_p,transient\_e\right\}$, where transient_p and transient_e are transient phases which are used to output platoon/revise messages and exit messages, respectively, by the intersection, empty indicates no platoon is entering the intersection (i.e., crossPlatoons=NULL), and cross indicates some platoons are entering the intersection (i.e., crossPlatoons!=NULL). The dynamic of evolution between them is shown in Figure 3.

When an intersection receives a platoon message of ($T_{head}$, $P_{size}$) from $i_{m}$, the platoon information along with the $i_{m}$ is added into crossPlatoons, and the time when the platoon reaches the corresponding exit segment $T_{head,e}$ is determined by adding a random delay $\delta_{I}$ (i.e., $T_{head,e}=T_{head}+\delta_{I}$). Then, the phase transits to transition_p in order to send out the platoon message of $\left(T_{head,e},P_{size}\right)$ to the exit segment (i.e., $ODMap(i_{m})$). Subsequently, the phase transits to cross immediately. In phase cross, if a platoon enters the intersection completely, the intersection removes the platoon from crossPlatoons. Then, if there are still platoons in crossPlatoons, the intersection remains cross. Otherwise, the phase jumps to empty.

When receiving a blocked exit message (i.e., exit=0) from $o_{n}$, the intersection moves to phase transient_e to send out blocked exit messages to the preselection lanes in $DOMap(o_{n})$. When receiving a free exit message (i.e., exit=1) from $o_{n}$, the intersection transits to phase transient_e to send free exit messages to the preselection lanes in ($DOMap(o_{n})\cap currentPhase$). If an external event occurs on port “tlc_in" and a phase index pi is obtained, the currentPhase is updated. Then, the intersection moves to transient_e to send out a free exit message to each preselection lane in TLPhases(pi) that is not blocked by the downstream segment and sends out a blocked exit message to each preselection lane in (Ent\TLPhases(pi)). In addition, if a platoon in crossPlatoons comes from the preselection lane $i_{m}$ in (Ent\TLPhases(pi)), it means the platoon is split by the red traffic light. As a result, a revise message is sent out to the exit segment $ODMap(i_{m})$ and the platoon information is removed from crossPlatoons.

## 3. Data Assimilation Framework for Vehicle Density Estimation Based on Particle Filters

In this section, we present the mesoscopic traffic data assimilation framework. Firstly, we formalize the state evolution based on the mesoscopic traffic model expressed in Section 2. Then, we describe the available traffic data and the measurement model which relates the measurement data to the system state. Subsequently, the particle filter for vehicle density estimation is presented. Finally, the weight computation method is illustrated based on the assumed error model of the noisy measurements.

### 3.1. The Evolution of Traffic State

According to the description of Section 2 and the formalization for discrete event state evolution in [30], the state of an urban traffic network can be defined as

(4) $$X_{\tilde{k}}=\left\{{\left\{\theta_{i,{\tilde{k}}_{i}},e_{i,{\tilde{k}}_{i}}\right\}}_{i\in \{So,Sg,Ag,Int,Tl,Sk\}},t_{\tilde{k}}\right\},\tilde{k}=0,1,\ldots ;{\tilde{k}}_{i}=0,1,\ldots ,$$

where $t_{\tilde{k}}$ is the time instant when the coupled network model transfers to the current state, $\theta_{i,{\tilde{k}}_{i}}$ represents the state of the atomic component *i*, $e_{i,{\tilde{k}}_{i}}$ is the elapsed time since the component *i* transfers to state $\theta_{i,{\tilde{k}}_{i}}$, and $\tilde{k}$ and ${\tilde{k}}_{i}$ are, respectively, the state index of the coupled model and atomic component *i*. As a result, we formalize the discrete event state evolution of an urban traffic network as

(5) $$X_{\tilde{k}}=TrafficSim\left(X_{\tilde{k}-1}\right)+\epsilon_{\tilde{k}-1},\tilde{k}=1,2,\ldots ,$$

where TrafficSim represents the platoon based traffic model, $\epsilon_{\tilde{k}-1}$ represents the system noise resulting from the randomness of atomic components.

### 3.2. Measurement Model

In this framework, the configurations of traffic signals in urban networks are assumed to be known, and sensors are deployed at inflow boundaries of some segments (an urban road is always subdivided into segments with small length in order to obtain an accurate traffic model, but it is difficult to deploy sensors that densely in the real traffic system). We assume that the sensors can detect and report vehicle passage times. The measurement data is available per time interval of length ΔT, and the measurements at the *k*-th interval are denoted as

(6) $$z_{k}=\left\{Y_{k}^{1},Y_{k}^{2},\ldots ,Y_{k}^{N_{s}}\right\},k=1,2,\ldots ,$$

where $N_{s}$ represents the number of sensors in an urban traffic network, and $Y_{k}^{i}$ is the vehicle passage times detected by the *i*-th sensor in the interval ((k−1)ΔT,kΔT]. The detections among sensors are considered independent and the measurement data is assumed to be noisy where both missed detection (i.e., the sensor fails to detect vehicle’s passage) and false detection (i.e., the sensor reports a passage when no vehicle passes by) exist. We define two parameters to model the two types of errors:

- detection accuracy *p*, representing the probability that a vehicle passage is detected by a sensor successfully. Consequently, the probability of a missed detection is 1−p.
- occurrence rate of false detection λ, indicating the number of false detections occurring in an unit time interval, which is assumed to be Poisson distributed.

In this framework, since passage times are related with the state transitions over the measurement interval, we formalize the measurement model as follows:

(7) $$z_{k}=h_{k}\left(X_{R_{k-1}+1:R_{k}}\right)+e_{k},k=1,2,\ldots ,$$

where $X_{R_{k-1}}$ is the state point retrieved at time (k−1)ΔT, $X_{R_{k-1}+1:R_{k}}$ represents a sequence of states indexed from $R_{k-1}+1$ to $R_{k}$ (i.e., state trajectory) which records the state transitions during ((k−1)ΔT,kΔT] completely, and $e_{k}$ is the measurement noise as is mentioned above.

### 3.3. Vehicle Density Estimation Using Particle Filters

#### 3.3.1. Principles of Particle Filters

Consider a general discrete state dynamic evolution as follows:

(8) $$\begin{array}{l} s_{0}∼p(\tilde{s_{0}}),\\ s_{k}=f_{k}\left(s_{k-1}\right)+\epsilon_{k-1},k=1,2,\ldots , \end{array}$$

where $p(\tilde{s_{0}})$ is the prior distribution, $s_{k-1}$, $s_{k}$ are respectively the state at time k−1 and *k*, $f_{k}$ is a possibly nonlinear function, and $\epsilon_{k-1}$ is a stochastic process noise. The measurement at time *k* is given by

(9) $$m_{k}=h_{k}\left(s_{k}\right)+e_{k},k=1,2,\ldots$$

in which $h_{k}$ is a possibly nonlinear function mapping the state $s_{k}$ to the measurement $m_{k}$, and $e_{k}$ is a measurement noise.

The particle filter aims to estimate the conditional probability density of all states up to time *k* based on all measurements until time *k*, that is, $p(s_{0:k}|m_{1:k})$, where $s_{0:k}=\left\{s_{0},s_{1},\ldots ,s_{k}\right\}$, $m_{1:k}=\left\{m_{1},m_{2},\ldots ,m_{k}\right\}$.

Assuming $p(s_{0:k-1}|m_{1:k-1}),$ the estimation at step k−1 is available, prediction step in Equation (10) and update step in Equation (11) are used to estimate the $p(s_{0:k}|m_{1:k})$ according to Bayes theorem [31]. In Equation (11), $p(s_{0:k}|m_{1:k-1})$ can be substituted with Equation (10) and $p(m_{k}|m_{1:k-1})$ is a normalizing constant. As a result, the sequential update is obtained in Equation (12). Note that $p\left(s_{k}|s_{0:k-1}\right)=p\left(s_{k}|s_{k-1}\right)$, $p\left(m_{k}|s_{0:k}\right)=p\left(m_{k}|s_{k}\right)$ according to the Markov property:

(10) $$p\left(s_{0:k}|m_{1:k-1}\right)=p\left(s_{k}|s_{0:k-1}\right)p\left(s_{0:k-1}|m_{1:k-1}\right),k=1,2\ldots ,$$

(11) $$p\left(s_{0:k}|m_{1:k}\right)=\frac{p\left(m_{k}|s_{0:k}\right)p\left(s_{0:k}|m_{1:k-1}\right)}{p(m_{k}|m_{1:k-1})},k=1,2,\ldots ,$$

(12) $$\begin{array}{cc} p(s_{0:k}|m_{1:k}) & =\frac{p\left(m_{k}|s_{0:k}\right)p\left(s_{k}|s_{0:k-1}\right)p\left(s_{0:k-1}|m_{1:k-1}\right)}{p(m_{k}|m_{1:k-1})},\\ & \propto p\left(m_{k}|s_{0:k}\right)p\left(s_{k}|s_{0:k-1}\right)p\left(s_{0:k-1}|m_{1:k-1}\right),k=1,2,\ldots \end{array}$$

Since it is always difficult to solve $p(s_{0:k}|m_{1:k})$ analytically, the particle filter approximates the $p(s_{0:k}|m_{1:k})$ with a set of Monte Carlo samples (particles) with their corresponding weights [32]. Let ${\left\{s_{0:k}^{i},w_{k}^{i}\right\}}_{i=1}^{N_{p}}$ represent the $p(s_{0:k}|m_{1:k})$, where $N_{p}$ is the particles size, $s_{0:k}^{i}$ is the *i*-th particle and $w_{k}^{i}$ is its weight. When the weights are normalized (i.e., $\sum_{i=1}^{N_{p}}w_{k}^{i}=1$), the $p\left(s_{0:k}|m_{1:k}\right)\approx \sum_{i=1}^{N_{p}}w_{k}^{i}\delta \left(s_{0:k}-s_{0:k}^{i}\right)$, where δ(x) is the Dirac delta distribution in vector form. Since it is usually intractable to draw from $p(s_{0:k}|m_{1:k})$ directly, the importance sampling method is employed in particle filters. In this method, if ${\left\{s_{0:k}^{i}\right\}}_{i=1}^{N_{p}}$ can be drawn from a probability $q(s_{0:k}|m_{1:k}),$ which is called importance density [32], then the weights ${\left\{w_{k}^{i}\right\}}_{i=1}^{N_{p}}$ are computed according to Equation (13):

(13) $$w_{k}^{i}=\frac{p(s_{0:k}^{i}|m_{1:k})}{q(s_{0:k}^{i}|m_{1:k})},k=1,2,\ldots$$

In recursive case, at step *k*, assuming that ${\left\{s_{0:k-1}^{i},w_{k-1}^{i}\right\}}_{i=1}^{N_{p}}$ characterizes the distribution of $p(s_{0:k-1}|m_{1:k-1})$ and the particles set ${\left\{s_{0:k-1}^{i}\right\}}_{i=1}^{N_{p}}$ is distributed according to $q(s_{0:k-1}|m_{1:k-1})$, then two steps are performed to generate ${\left\{s_{0:k}^{i},w_{k}^{i}\right\}}_{i=1}^{N_{p}}$ characterizing $p(s_{0:k}|m_{1:k})$:

- augment each particle $s_{0:k-1}^{i}$ with sample $s_{k}^{i}∼q\left(s_{k}|s_{0:k-1}^{i},m_{1:k}\right)$ to form $s_{0:k}^{i}∼q\left(s_{0:k}|m_{1:k}\right)$, where $q\left(s_{0:k}|m_{1:k}\right)=q\left(s_{k}|s_{0:k-1},m_{1:k}\right)q\left(s_{0:k-1}|m_{1:k-1}\right)$;
- update weights by
  (14) $$w_{k}^{i}=\frac{p(s_{0:k}^{i}|m_{1:k})}{q(s_{0:k}^{i}|m_{1:k})}\propto \frac{p\left(m_{k}|s_{0:k}^{i}\right)p\left(s_{k}^{i}|s_{0:k-1}^{i}\right)p\left(s_{0:k-1}^{i}|m_{1:k-1}\right)}{q\left(s_{k}^{i}|s_{0:k-1}^{i},m_{1:k}\right)q\left(s_{0:k-1}^{i}|m_{1:k-1}\right)}=\frac{p\left(m_{k}|s_{k}^{i}\right)p\left(s_{k}^{i}|s_{k-1}^{i}\right)}{q(s_{k}^{i}|s_{0:k-1}^{i},m_{1:k})}w_{k-1}^{i}.$$

The system transition density is a common choice of the importance density, namely, $q\left(s_{k}|s_{0:k-1},m_{1:k}\right)=p\left(s_{k}|s_{k-1}\right)$. Consequently, Equation (14) is simplified to

(15) $$w_{k}^{i}=p\left(m_{k}|s_{k}^{i}\right)w_{k-1}^{i},k=1,2,\ldots$$

In the particle filter, degeneracy phenomenon is a common problem which means most particles have negligible weights and the effective particle set is reduced to very few particles after a few iterations. In order to reduce the influence of the degeneracy, a resampling step is performed after the particles are updated.

#### 3.3.2. Particle Filtering for Vehicle Density Estimation

It has been proven that the variable dimensions of both the system state and the discrete event state trajectory have no tangible effect on the updating of particles and their weights in particle filters by previous studies [24,30,33]. Therefore, we can safely apply the particle filter to estimate vehicle densities in our study. Since we map the traffic state trajectory during the measurement interval to the vehicle passage times in the measurement model of Equation (7), the particle weight should be updated as

$$w_{k}=p\left(z_{k}|X_{R_{k-1}+1:R_{k}}\right)w_{k-1},k=1,2,\ldots$$

Algorithm 1 describes the main steps to estimate traffic densities using particle filters.

**Algorithm 1:** The particle filter for vehicle density estimation

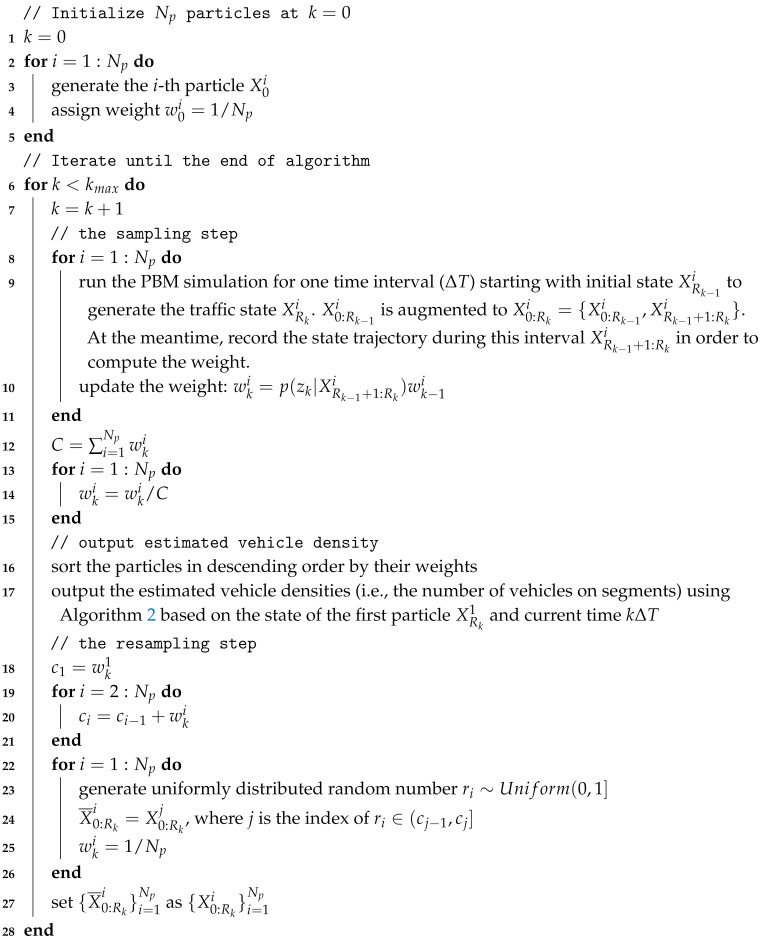



At step k=0, we randomly generate $N_{p}$ particles by guessing the size, position, and speed of platoons over the network, and all weights are initialized to $1/N_{p}$ (lines 2–5). Then, the following steps are iterated until the end of algorithm:

- Sampling step: for each particle, we run the mesoscopic traffic simulation for ΔT, the particle is updated and the state trajectory over this interval is recorded. Then, the particle’s weight is calculated based on (noisy) newly available passage times and the recorded state trajectory (the method of weight computation is depicted in Section 3.3.3). After all particles are updated, the normalization of the weights is performed to prepare for resampling (lines 8–15).
- Output step: we obtain the estimated vehicle densities (i.e., the number of vehicles on segments) from the state of the particle with the highest weight (lines 16–17). The number of vehicles on a segment is calculated by excluding the vehicles which have not entered or have left the segment from vn, and the detailed process is illustrated in Algorithm 2.
- Resampling step: we resample the newly generated particles by replicating particles in proportion to their weights (lines 18–27).

**Algorithm 2:** Calculating the number of vehicles on segments based on the traffic state

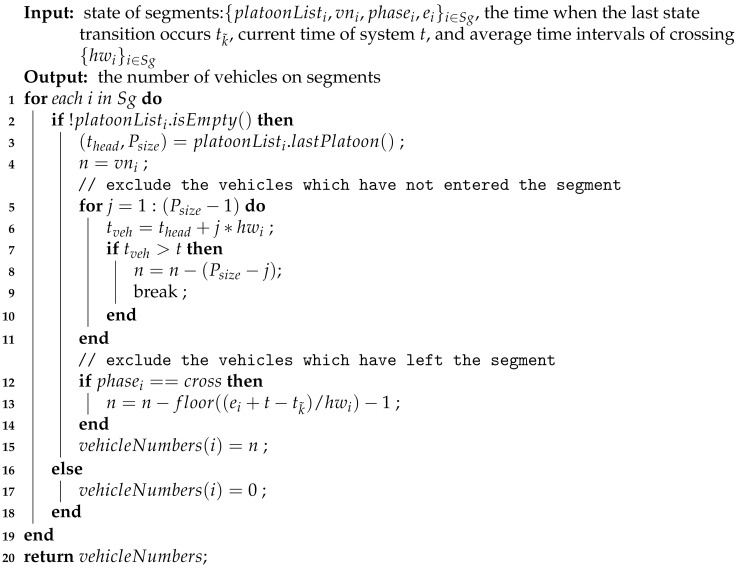



#### 3.3.3. Weight Computation

When a sample is generated, the state trajectory is recorded (i.e., $X_{R_{k-1}+1:R_{k}}^{i}$), newly available measurement and the error model are used to compute $p(z_{k}|X_{R_{k-1}+1:R_{k}}^{i})$, where $z_{k}=\left\{Y_{k}^{1},Y_{k}^{2},\ldots ,Y_{k}^{N_{s}}\right\}$. Since all sensors detect vehicle passages independently, we have

(16) $$p\left(\left\{Y_{k}^{1},Y_{k}^{2},\ldots ,Y_{k}^{N_{s}}\right\}|X_{R_{k-1}+1:R_{k}}^{i}\right)=\begin{array}{c} \prod_{j=1}^{N_{s}}p\left(Y_{k}^{j}|X_{R_{k-1}+1:R_{k}}^{i}\right). \end{array}$$

In order to compute $p(Y_{k}^{j}|X_{R_{k-1}+1:R_{k}}^{i})$, we obtain the estimated passage times at the *j*-th sensor (denoted as ${\tilde{Y}}_{k}^{i,j}$) from $X_{R_{k-1}+1:R_{k}}^{i}$, and, as a result, $p\left(Y_{k}^{j}|X_{R_{k-1}+1:R_{k}}^{i}\right)=p\left(Y_{k}^{j}|{\tilde{Y}}_{k}^{i,j}\right)$. Then, a match procedure [24] is employed to define missed detections and false detections based on the measurement $Y_{k}^{j}$ and the estimated value ${\tilde{Y}}_{k}^{i,j}$. Lastly, according to the error model depicted in Section 3.2, $p(Y_{k}^{j}|{\tilde{Y}}_{k}^{i,j})$ is computed by

(17) $$p\left(Y_{k}^{j}|{\tilde{Y}}_{k}^{i,j}\right)=p^{n_{i,j}-n_{m}}{\left(1-p\right)}^{n_{m}}\times \frac{{\left(\lambda \Delta T\right)}^{n_{o}}e^{-\lambda \Delta T}}{n_{o}!}\times e^{-d_{m}},$$

where $n_{i,j}$ is the number of passage times in ${\tilde{Y}}_{k}^{i,j}$, $n_{m}$ is the number of missed detections, and $n_{o}$ is the number of false detections. The term $p^{n_{i,j}-n_{m}}{\left(1-p\right)}^{n_{m}}$ represents the probability of missed detection errors, and the term $\frac{{\left(\lambda \Delta T\right)}^{n_{o}}e^{-\lambda \Delta T}}{n_{o}!}$ represents the probability of false detection errors, and $e^{-d_{m}}$ is a penalty term where $d_{m}$ is the maximum distance in all matched pairs. More details about the math procedure can be found in [24].

## 4. Experiments

### 4.1. Experimental Design

**Figure 4:**
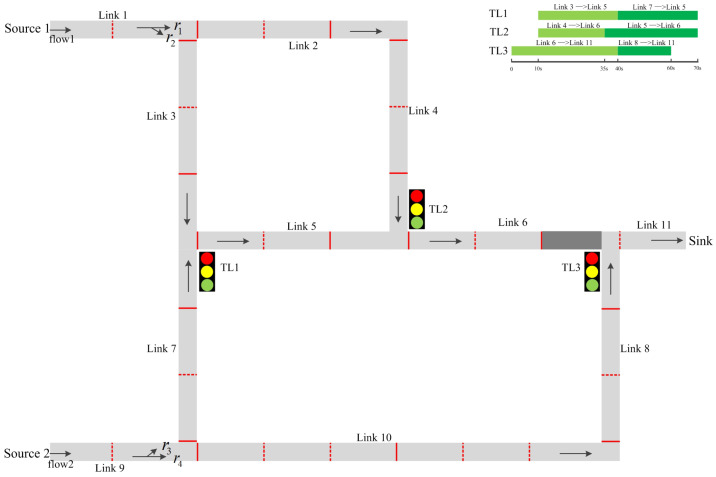
The urban traffic network used in the experiments.

The urban traffic network used in the experiments is shown in Figure 4, where 11 links are connected by seven intersections. In this network, two source nodes generate platoons traveling to the sink, where the mean time gap between successive platoons is 8 s (minimum time gap is 5 s, the mean of random time gap is 3 s) and the average time interval of crossing a boundary is 1.2 s. Platoons are always able to exit the network from the sink. Each link is subdivided into road segments with lengths of 100 m, and 16 sensors are regularly deployed in the network (both the red solid line and the red dotted line represent the inflow boundary of a road segment. The red solid line also indicates the place where a sensor is deployed). All road segments have a speed limit of 15 m/s and a capacity of 16 vehicles. Three fixed time traffic lights with a cycle length of 60 s are used to control the conflicting movements at intersections. The offset and duration of the green lights for each movement at traffic lights are shown at the right top of Figure 4. At the end of link 1 and link 9, platoons are split and assigned to different exit links according to the turning probabilities of $r_{1}$, $r_{2}$ and $r_{3}$, $r_{4}$, respectively.

Firstly, a simulation of the urban traffic network is performed, and all data is recorded. The simulation is considered as the real system, and the recorded data is regarded as the ground truth data. Then, the ground truth data are processed based on the assumed error model to produce the noisy measurement data that will be used in the data assimilation to estimate the vehicle density.

Then, we build an imperfect traffic model by adding errors in the model parameters (see Table 2). The traffic network is simulated again using the imperfect traffic model to get the estimation without data assimilation (we refer these results as the simulated results). Next, the real measurements from the real system are assimilated into the imperfect traffic model to generate the estimation with data assimilation (we refer these results as the filtered results). By assimilating the noisy measurement data, the filtered results are expected to be more accurate than the simulated results.

Specifically, two cases are tested in our experiments where the vehicle arrival rates of the network (represented by flow1 and flow2 in Figure 4) and the turning probabilities at intersections (represented by $r_{1}$, $r_{2}$ and $r_{3}$, $r_{4}$ in Figure 4) are perturbed to get the imperfect traffic models, respectively. The configuration of these parameters is illustrated in Table 2. In the real system, an average of 1000 vehicles per hour enter the network from source 1 and 1200 vehicles per hour enter from source 2. A vehicle reaching the exit point of link 1 moves to link 2 and link 3 with probability of 0.4 and 0.6, respectively, at the end of link 9, these probabilities are 0.6 to link 7, and 0.4 to link 10. In case 1, the imperfect traffic model has inaccurate vehicle arrival rates. Specifically, the flow from source 1 is 200 vehicles per hour more than the real flow, while the flow from source 2 is 200 vehicles per hour less than the real flow. In case 2, the turning probabilities are erroneous in the imperfect traffic model. The probability of traveling to link 2 and link 3 from link 1 is set to 0.6 and 0.4, respectively, while the probability of traveling to link 7 and link 10 from link 9 is set to 0.4 and 0.6, respectively.

We implement an event scheduling based discrete event simulator using c++ on which we run our simulation model. A simulation of 1200 s is considered in all experiments and the number of vehicles on the segments in the urban network are recorded every 60 s. We run the real system for 120 s as a warm-up period. The initial network states of the particles are randomly sampled based on the real network state at 120 s, and the results of 18 cycles (from 180 s to 1200 s) are used to evaluate the effectiveness of the data assimilation framework. In the data assimilation system, the noisy measurement data (i.e., vehicle passage times at each sensor in this network) are available every 60 s.

### 4.2. Evaluation Criteria

In this section, the measurement error model is fixed with detection accuracy p=0.9, occurrence rate of false detection λ = 1/300 s^−1^, and 1000 particles are employed in the data assimilation system. The goal of our experiments is twofold: we intend to show that the filtered results are more accurate than the simulated results when compared with the ground truth, and we want to explore whether the filtered results can estimate the ground truth accurately.

In order to quantify the proximity between two traffic states, we consider the Root Mean Square Error (RMSE) of the number of vehicles on segments as the evaluation criteria, that is,

(18) $$RMSE_{e,k}=\sqrt{\frac{\sum_{i=1}^{N_{s}}{\left({s^{e}}_{i,k}-{s^{r}}_{i,k}\right)}^{2}}{N_{s}}},$$

where ${RMSE}_{e,k}$ represents the RMSE of the estimated results (including the simulated results and the filtered results) comparing with the ground truth at time step *k*, $N_{s}$ is the total number of segments in the traffic network, ${s^{r}}_{i,k}$ indicates the number of vehicles on the *i*-th segment in the ground-truth traffic state at time instant kΔT while ${s^{e}}_{i,k}$ is the corresponding number in the estimated state at the same time instant.

In order to illustrate the accuracy of the estimation to the ground truth, we base our analysis on the estimation results of the vehicle density on an arbitrarily selected road segment. In our case, the 17th road segment (the one in dark in Figure 4) is chosen.

### 4.3. Experimental Results

#### 4.3.1. Test Case 1

**Figure 5:**
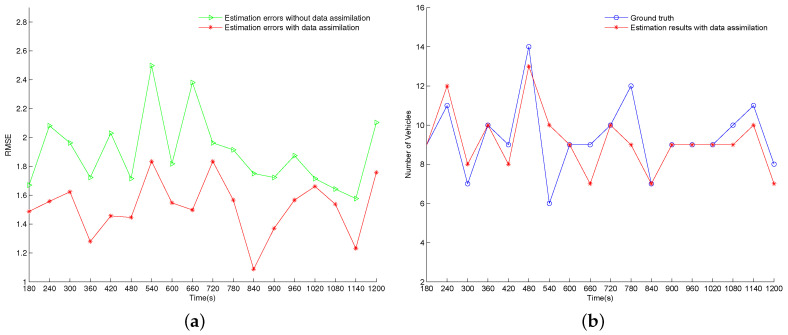
Experimental results of case 1. (**a**) RMSE results; (**b**) The estimated number of vehicles on the 17th road segment with data assimilation.

Figure 5 displays the experimental results of test case 1 where the vehicle arrival rates are inaccurate. Figure 5a shows the RMSE errors of the estimation results with and without data assimilation, respectively. As shown in the figure, the RMSE errors of the estimation results with data assimilation are smaller than that of the estimation results without data assimilation at all time steps, which indicates that the data assimilation framework has improved the estimation results of the whole traffic network with the help of the sensor data. The RMSE errors of the estimation results without data assimilation are decreased by an average of 19.4% when the noisy measurement data are assimilated using the proposed data assimilation framework. Figure 5b compares the estimated number of vehicles using data assimilation (blue line) with the ground-truth value (red line) on the 17th road segment. From the figure, we can see that the estimated number follows the real number at most of the time steps. The Mean Absolute Percentage Error (MAPE) of the estimated number over 18 cycles is 10.4%, which indicates a promising performance. At some points (for example, t = 540 s, 660 s, 780 s), the estimated numbers contain relatively large errors. One possible reason is that only the most likely particle is insufficient to represent the whole possibility distribution. Future research is needed to find a suggestion that can better reflect the “belief histogram" estimated by particle filters.

#### 4.3.2. Test Case 2

**Figure 6:**
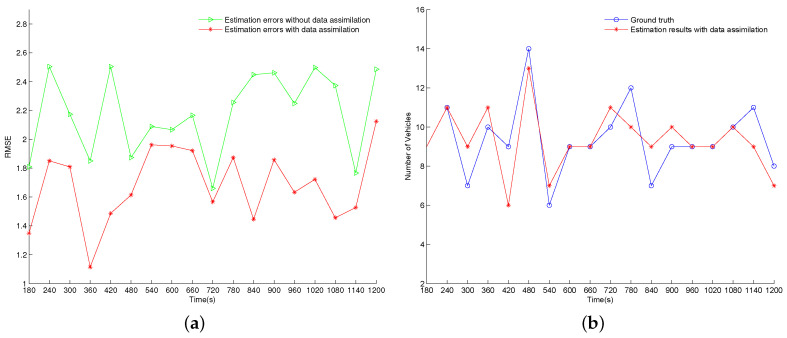
Experimental results of case 2. (**a**) RMSE results; (**b**) The estimated number of vehicles on the 17th road segment with data assimilation.

This case examines the effectiveness of this proposed data assimilation framework dealing with the erroneous turning probabilities. The experimental results are displayed in Figure 6. From Figure 6a, we can see that the RMSE errors of the estimation results without data assimilation are larger than that in case 1 on the whole. It indicates an increasing challenge of estimating the ground truth. Similar to the experimental results in case 1, this data assimilation framework reduces the RMSE errors of estimation results at all time steps by assimilating the sensor data in this case. After assimilating the noisy measurement data using the proposed data assimilation framework, the RMSE errors of the estimation results without data assimilation are reduced by 21.1% on average. Figure 6b shows the estimated number of vehicles with data assimilation and the ground-truth value on the 17th segment. The MAPE over 18 cycles is 10.7% in this case, which exhibits a comparable effectiveness to that in test case 1.

### 4.4. Sensitivity Analysis

In this section, a series of additional experiments are carried out to analyze the sensitivity of the estimation results to several key factors of the proposed data assimilation framework. These factors include the measurement data quality and the number of particles. The average RMSE error over 18 cycles is used to quantify the experimental results:

(19) $$\bar{RMSE}=\frac{\sum_{k=1}^{N_{cycles}}RMSE_{k}}{N_{cycles}}.$$

For each combination of parameters, we present the average result of 10 independent experiments.

#### 4.4.1. Effect of Measurement Data Quality

**Figure 7:**
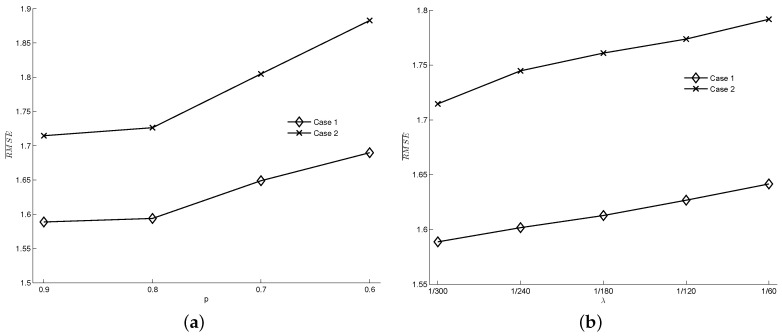
The influence of sensor data quality on data assimilation results. (**a**) The effect of *p* (λ = 1/300 s^−1^, $N_{p}=1000$); (**b**) The effect of λ (p=0.9, $N_{p}=1000$).

In the measurement model of this study, detection accuracy *p* and occurrence rate of false detection λ characterize the quality of the noisy data. Therefore, we explore the effect of sensor quality by varying *p* and λ. The set of parameters used in the case experiments (i.e., $N_{p}=1000$, p=0.9, λ = 1/300 s^−1^) are selected as the baseline. When varying *p*, we remain λ = 1/300 s^−1^; when varying λ, we keep p=0.9. The results are shown in Figure 7a,b, respectively. Coinciding with our expectations, in both cases, the data assimilation performance deteriorates as the data quality becomes worse. However, even when the detection accuracy falls to 0.6 or the false rate increases to 1/60 s^−1^, the performance is still better than that of the estimation results without data assimilation (in test case 1 and case 2, the $\bar{RMSE}$ of the estimation results without data assimilation are 1.86 and 2.15, respectively), which indicates the robustness of this framework to measurement data errors.

#### 4.4.2. Effect of the Number of Particles

**Figure 8:**
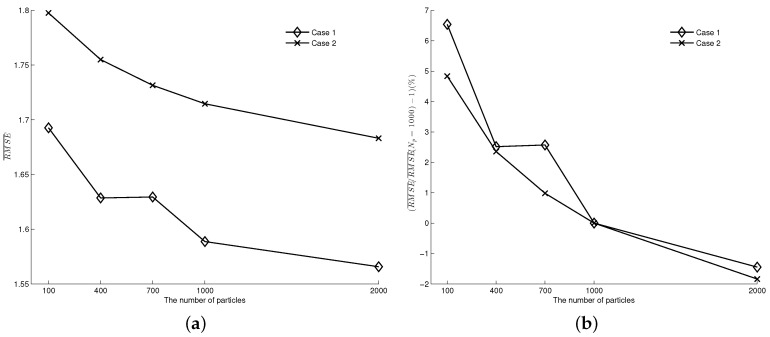
The influence of number of particles on data assimilation results (p=0.9,λ = 1/300 s^−1^). (**a**) $\bar{RMSE}$ error; (**b**) The increased percentage of $\bar{RMSE}$ relative to that at $N_{p}=1000$.

We fix p=0.9, λ = 1/300 s^−1^ in both cases and vary the number of particles used in the algorithm from 100 to 2000. The results are displayed in Figure 8a. From the figure, we can see that, as the number of particles increases from 100 to 2000, the $\bar{RMSE}$ error decreases in both cases. The more particles used, the better the performance. However, we note that the decrease of $\bar{RMSE}$ error is not proportional to the increase of the number of particles. Figure 8b shows the increased percentage of $\bar{RMSE}$ error relative to that at 1000 particles (i.e., ($\bar{RMSE}/\bar{RMSE}\left(N_{p}=1000\right)-1)$). The plot tells that a reduction from 1000 to 100 leads to an increase of about 5.6% (6.54% in case 1, 4.83% in case 2) of the error measure, while doubling the number of particles improves the performance about 1.6% (1.44% in case 1, 1.84% in case 2).

## 5. Conclusions

In this study, we presented a data assimilation framework for vehicle density estimation on urban traffic networks. In this data assimilation framework, a mesoscopic traffic model (i.e., platoon based model) was employed since it is not only able to capture more details compared with macroscopic traffic models, but also has the advantage of computing faster than microscopic traffic models. The passage times of individual vehicle were considered as the measurement data, which contains errors of missed and false detection. Since the mesoscopic traffic model is nonlinear and the vehicle passage times contain strongly non-Gaussian noises, particle filters, which impose no restriction on the model dynamics and error assumptions, were applied to conduct the data assimilation.

In order to test this data assimilation framework, we conducted experiments in a simulated urban traffic network. Experimental results show that the proposed data assimilation framework can provide more accurate estimation results compared to those produced without data assimilation. More specifically, the average percentage of reduced errors of 19.4% and 22.1% are achieved in the two test cases (one with errors in vehicle arrival rates, and the other with errors in turning probabilities), respectively. With regard to the estimation accuracy, the estimated results are able to follow the real situation at most time steps. The absolute percentage errors of the estimated vehicle density are respectively 10.4% and 10.7% in the two cases, which indicates a promising performance.

Sensitivity analysis indicates that this data assimilation framework is robust to both measurement errors and model errors. In both cases, even with 40% missed passage times or one false detection per minute, the performance does not deteriorate too much and is still superior to that without data assimilation. It is noticed that the improvement of performance is not proportional to the increase of the number of particles. Specifically, an increase of the number of particles from 1000 to 2000 leads to an improvement of about 1.6%, while a reduction of the number of particles from 1000 to 100 results in a deterioration of about 5.6%.

Future research directions include looking for an appropriate real-life scenario to further evaluate and apply the data assimilation framework, and integrating traffic data from different sources (for example, route choice fractions from automated vehicle identification (AVI) system and travel time from floating-car data) in the data assimilation framework to improve the accuracy and robustness of the estimation results further. Another direction is to combine the state estimation framework with urban traffic control strategies to improve the performance of the urban traffic networks.

## Figures and Tables

**Table 1:** Phases and state variables of key atomic models of the urban traffic system.

Model Type	Phases	State Variables	Description
Source	active	p_time	The time when sending a platoon message
		p_size	The number of vehicles within the generated platoon
Segment	empty approach		
	cross blocked	platoonList	The container of the information of all platoons on the segment (the platoon that is entering or leaving the segment is also in it)
	blocked_in	vn	The number of all vehicles in platoonList
	blocked_full	out	The state of the exit boundary of the segment (blocked or free)
	transient_p transient_e		
Intersection	empty		
	cross transient_p	crossPlatoons	The container of related information of platoons which are entering the intersection
	transient_e	currentPhase	The current phase of the traffic light in the intersection

**Table 2:** The configuration of the perturbed parameters in experiments.

	flow1 (vehs/hour)	flow2 (vehs/hour)	$r_{1}$	$r_{2}$	$r_{3}$	$r_{4}$
Perfect parameters	1000	1200	0.4	0.6	0.6	0.4
Imperfect parameters (Case 1)	1200	1000	0.4	0.6	0.6	0.4
Imperfect parameters (Case 2)	1000	1200	0.6	0.4	0.4	0.6
